# Supplementary material for: Metabolic Rewiring and Altered Glial Differentiation in an iPSC-Derived Astrocyte Model Derived from a Nonketotic Hyperglycinemia Patient
Source: Int J Mol Sci. 2024 Feb 28;25(5):2814. doi: 10.3390/ijms25052814 (PMC10931996; doi:10.3390/ijms25052814)
Supplement: Supplementary file 1 [file ijms-25-02814-s001.zip › Annex 1_L.pdf]

Annex 1: Genetic background of the iPSCs

Whole exome sequencing has been performed in both iPSCs lines (control and GLDC27-iPS4F-1). The results included in this annex refer to what was detected in the GLDC27-iPS4F-1 iPSCs line. The results were analyzed using SOPHIA Genetics' WESv2 sequencing panel and the SOPHIA DDM platform.

Coverage statistical data

25x	50x	100x	200x	500x	1000x	Coverage 10% quartile	Coverage Heterogeneity
99.45%	98.14%	83.08%	20.06%	0.15%	0.06%	85×	0.72%

Filtering and prioritizing variants in the index case:

229458 **variants** have been obtained in 19425 **genes**.
Filtering of variants with a frequency < 1% according to GnomAD and G1000 (37009). Frequency in the Community and Career (19631). VF>25% (9253). *Depth>10 (8233).*

A) **Freq/PathScore/Conseq**: ACMG score P, LP (28). SOPHiA score A, B (19). Non ClinVar benign (19). Nonsynonymous, non-intronic, non-UTR (19). Retained Variants (14). Prioritization of Variants in Genes Related to Metabolic Diseases***** (2 – *ALOX12B* y *SGMS2*).

B) **Freq/PathVUSScore/Conseq**: ACMG score P, LP y VUS (8140). SOPHiA score A, B y C (8140). Non ClinVar benign (8033). Nonsynonymous, non-intronic, non-UTR (570). Retained Variants (538). Prioritization of Variants in Genes Related to Metabolic Diseases***** (27). Flagging Bening (20).
   a. SIFT/PolyPhen2 damaging (11).

C) **Ser-Gly Metabolism***: Filtering of variants with a frequency < 1% according to GnomAD and G1000 (8). Community frequency and career (4). VF>25% (1). Depth>10 (1). ACMG score P, LP y VUS (1). SOPHiA score A, B y C (1). No ClinVar benign (1). Likely Bening (0).

D) **Neural Differentiation Markers****: Filtering of variants with a frequency < 1% according to GnomAD and G1000 (17). Frequency in the community and career (10). VF>25% (2). Depth>10 (2). ACMG score P, LP y VUS (2). SOPHiA score A, B y C (2). Non ClinVar benign (2). Likely Bening (0).

E) **Cyclins and Creatine Metabolism *****: Filtering of variants with a frequency < 1% according to GnomAD and G1000 (1). Frequency in the community and career (1). VF>25% (1). Depth>10 (1). ACMG score P, LP y VUS (1). SOPHiA score A, B y C (1). Non ClinVar benign (1). Likely Bening (0).

F) **MICOS Complex ******: Filtering of variants with a frequency of < 1% according to GnomAD and G1000 (15). Frequency in the community and career (10). VF>25% (3).

Depth>10 (3). ACMG score P, LP y VUS (3). SOPHiA score A, B y C (3). Non ClinVar benign (3). Likely Bening (0).

G) **SOPHiA Prediction:** ACMG score P, LP, VUS (8140). SOPHiA score A, B (210). Non ClinVar benign (185). Nonsynonymous, non-intronic, non-UTR (184). Retained Variants (176). SOPHiA Path (3).

H) **CNV:** rejected. Visually looking at the genes related to neuronal differentiation markers it appears that *NES, GFAP* would have a duplication of the entire gene (also observed in the control iPSCs line).

I) **mtDNA:** 52 variants. Low Frequency (11). Not synonymous or intergenic (2). Non mitomap/Bening/Flagging variant (0).

* **Ser-Gly Metabolism Genes (6):** *PHGDH PSAT1 PSPH SHMT1 SHMT2 SRR*

** **Neural Differentiation Markers Genes (11):** *PAX6 NES SOX1 MAP2 FOXG1 SOX17 TBXT SLC2A1 SLC1A3 GFAP S100B*

*** **Other Genes (4):** *GCAT GATM CCND1 CDK2*

**** **MICOS Complex Genes:** *APOO APOOL CHCHD3 CHCHD6 DNM1L IMMT MFN1 MFN2 OPA1*

***** **ICIMD Genes (1382):** *A4GALT AAGAB AARS1 AARS2 AASS ABAT ABCA1 ABCA12 ABCB11 ABCB4 ABCB6 ABCB7 ABCC2 ABCC6 ABCC8 ABCD1 ABCD3 ABCD4 ABCG5 ABCG8 ABHD12 ABHD5 ACACA ACACB ACAD8 ACAD9 ACADM ACADS ACADSB ACADVL ACAT1 ACAT2 ACBD5 ACER3 ACO2 ACOX1 ACOX2 ACSF3 ACSL4 ACY1 ADA ADA2 ADAR ADARB1 ADAT3 ADCK2 ADK ADSL ADSS1 AFG3L2 AGA AGK AGL AGMO AGPAT2 AGPS AGXT AGXT2 AHCY AICDA AIFM1 AIMP1 AIMP2 AK1 AK2 AK7 AKR1C2 AKR1D1 AKT2 ALAD ALAS2 ALB ALDH18A1 ALDH1L2 ALDH3A2 ALDH4A1 ALDH5A1 ALDH6A1 ALDH7A1 ALDOA ALDOB ALG1 ALG11 ALG12 ALG13 ALG14 ALG2 ALG3 ALG6 ALG8 ALG9 ALOX12B ALOXE3 ALPI ALPL AMACR AMN AMPD1 AMPD2 AMPD3 AMT ANGPTL3 ANPEP AP1B1 AP1S1 AP1S2 AP2S1 AP3B1 AP3B2 AP3D1 AP4B1 AP4E1 AP4M1 AP4S1 AP5Z1 APOA1 APOA5 APOB APOC2 APOC3 APOE APOO APOPT1 APPL1 APRT AR ARCN1 ARFGEF2 ARG1 ARSA ARSB ARSG ASAH1 ASL ASNS ASPA ASS1 ATAD1 ATAD3A ATG5 ATIC ATP13A2 ATP5F1A ATP5F1D ATP5F1E ATP5MD ATP5PO ATP6AP1 ATP6AP2 ATP6V0A2 ATP6V1A ATP6V1E1 ATP7A ATP7B ATP8A2 ATP8B1 ATPAF2 AUH B3GALNT2 B3GALT6 B3GAT3 B3GLCT B4GALNT1 B4GALT1 B4GALT7 B4GAT1 BAAT BBOX1 BCAP31 BCAT2 BCKDHA BCKDHB BCKDK BCS1L BLK BLOC1S3 BLOC1S6 BLVRA BMP6 BMS1 BOLA3 BPNT2 BSCL2 BTD C12orf65 C1GALT1C1 C1QBP CA5A CAD CANT1 CARS1 CARS2 CAT CBLIF CBS CCDC115 CCS CD320 CEP89 CERS1 CERS2 CERS3 CETP CHAT CHCHD10 CHCHD2 CHKB CHRNE CHST11 CHST14 CHST3 CHST6 CHSY1 CLCN2 CLN3 CLN5 CLN6 CLN8 CLP1 CLPB CLPP CLPX CLTC CMPK2 CNDP1 COA3 COA5 COA6 COA7 COASY COG1 COG2 COG4 COG5 COG6 COG7 COG8 COL4A3BP COPA COPB2 COQ2 COQ4 COQ5 COQ6 COQ7 COQ8A COQ8B COQ9*

COX10 COX14 COX15 COX20 COX4I1 COX4I2 COX5A COX6A1 COX6A2 COX6B1 COX7B COX8A CP CPOX CPS1 CPT1A CPT1C CPT2 CRAT CRPPA CSGALNACT1 CTH CTNS CTPS1 CTSA CTSB CTSC CTSD CTSF CTSK CUBN CYB561 CYB5A CYB5R3 CYC1 CYCS CYP11A1 CYP11B1 CYP11B2 CYP17A1 CYP19A1 CYP21A2 CYP27A1 CYP2U1 CYP4F22 CYP51A1 CYP7A1 CYP7B1 D2HGDH DALRD3 DARS1 DARS2 DBH DBT DCXR DDC DDHD1 DDHD2 DDOST DEGS1 DGAT1 DGKE DGUOK DHCR24 DHCR7 DHDDS DHFR DHODH DHTKD1 DIABLO DKC1 DLAT DLD DLST DMGDH DNA2 DNAJC12 DNAJC19 DNAJC21 DNAJC5 DNAJC6 DNM1 DNM1L DNM2 DOLK DPAGT1 DPM1 DPM2 DPM3 DPYD DPYS DSE DTNBP1 DTYMK DUT DYM DYNC1H1 EARS2 EBP ECHS1 EFL1 EHHADH EIF6 ELAC2 ELOVL1 ELOVL4 ELOVL5 ELP1 ELP2 EMC1 EMG1 ENO3 ENPP1 ENTPD1 EOGT EPG5 EPHX1 EPM2A EPRS1 ERAL1 ESR1 ESR2 ETFA ETFB ETFDH ETHE1 EXT1 EXT2 EXTL3 FA2H FAAH2 FAH FAM20B FAR1 FARS2 FARSA FARSB FASTKD2 FBP1 FBXL4 FDFT1 FDPS FDX2 FDXR FECH FH FIG4 FKRP FKTN FLAD1 FMO3 FOLR1 FOXRED1 FTCD FTH1 FTL FTSJ1 FUCA1 FUK FUT8 FXN G6PC G6PC3 G6PD GAA GABBR2 GABRA1 GABRA6 GABRB1 GABRB2 GABRB3 GABRD GABRG2 GAD1 GALC GALE GALK1 GALM GALNS GALNT14 GALNT3 GALT GAMT GANAB GARS1 GATA1 GATB GATC GATM GBA GBA2 GBE1 GCDH GCH1 GCK GCLC GDAP1 GFER GFM1 GFM2 GFPT1 GGCX GGPS1 GGT1 GK GLA GLB1 GLDC GLRA1 GLRB GLRX5 GLS GLUD1 GLUL GLYCTK GM2A GMPPA GMPPB GNE GNMT GNPAT GNPNAT1 GNPTAB GNPTG GNS GON7 GORAB GOSR2 GOT2 GPAA1 GPD1 GPHN GPI GPIHBP1 GPT2 GPX4 GRHPR GRIA2 GRIA3 GRIA4 GRID2 GRIN1 GRIN2A GRIN2B GRIN2D GRM1 GRM6 GRN GSR GSS GSTZ1 GTPBP3 GUF1 GUSB GYG1 GYG2 GYS1 GYS2 H6PD HAAO HACD1 HADH HADHA HADHB HAL HAMP HAO1 HARS1 HARS2 HCCS HCFC1 HEPHL1 HEXA HEXB HFE HGD HGSNAT HIBADH HIBCH HJV HK1 HLCS HMBS HMGCL HMGCS2 HMOX1 HNF1A HNF1B HNF4A HOGA1 HPD HPGD HPRT1 HPS1 HPS3 HPS4 HPS5 HPS6 HS6ST1 HS6ST2 HSD11B1 HSD11B2 HSD17B10 HSD17B3 HSD17B4 HSD3B2 HSD3B7 HSPA9 HSPD1 HSPE1 HTRA2 HYAL1 HYKK IARS1 IARS2 IBA57 IDH1 IDH2 IDH3A IDH3B IDS IDUA IFIH1 IL1RAPL1 IMPDH1 INPP5E INPP5K INPPL1 INS INSR ISCA1 ISCA2 ISCU ITPA ITPR1 ITPR2 IVD JAGN1 KARS1 KCNJ11 KCTD7 KDSR KHK KIF1A KIF5A KIF5C KLF11 KMO KYNU L2HGDH LACC1 LAGE3 LAMP2 LAP3 LARGE1 LARS1 LARS2 LBR LCAT LCT LDHA LDHB LDHD LDLR LDLRAP1 LFNG LIAS LIPA LIPC LIPE LIPH LIPT1 LIPT2 LMAN1 LMBRD1 LMF1 LONP1 LPA LPAR6 LPIN1 LPIN2 LPL LRPPRC LRRK2 LSS LTC4S LYRM4 LYRM7 LYST MAGT1 MAN1B1 MAN2B1 MAN2B2 MANBA MAOA MAOB MARS1 MARS2 MAT1A MAT2A MBOAT7 MBTPS1 MC2R MCAT MCCC1 MCCC2 MCEE MCFD2 MCOLN1 MDH1 MDH2 MECR MFF MFN2 MFSD2A MFSD8 MGAT2 MGME1 MICOS13 MICU1 MICU2 MIEF2 MIPEP MLPH MLYCD MMAA MMAB MMACHC MMADHC MMUT MOCOS MOCS1 MOCS2 MOCS3 MOGS MPC1 MPDU1 MPI MPST MPV17 MRAP MRM2 MRPL12 MRPL24 MRPL3 MRPL44 MRPS14 MRPS16 MRPS2 MRPS22 MRPS23 MRPS25 MRPS28 MRPS34 MRPS7 MSMO1 MSTO1 MT-ATP6 MT-ATP8 MT-CO1 MT-CO2 MT-CO3 MT-CYB MT-ND1 MT-ND2 MT-ND3 MT-ND4 MT-ND4L MT-ND5 MT-ND6 MT-RNR1 MT-RNR2 MT-TA MT-TC MT-TD MT-TE MT-TF MT-TG MT-TH MT-TI MT-TK MT-TL1 MT-TL2 MT-TM MT-TN MT-TP MT-TQ MT-TR MT-TS1 MT-TS2 MT-TT MT-TV MT-TW MT-TY MTAP MTFMT MTHFD1 MTHFR MTHFS MTM1 MTMR2 MTO1 MTPAP MTR MTRR MTTP MVD MVK MYO5A NADK2 NADSYN1 NAGA NAGLU NAGS NANS NAPB NARS1 NARS2 NAT8L NAXD NAXE NBAS NBEAL2 NDST1 NDUFA1 NDUFA10 NDUFA11 NDUFA12 NDUFA13 NDUFA2

NDUFA4 NDUFA6 NDUFA8 NDUFA9 NDUFAF1 NDUFAF2 NDUFAF3 NDUFAF4 NDUFAF5 NDUFAF6 NDUFAF7 NDUFAF8 NDUFB10 NDUFB11 NDUFB3 NDUFB8 NDUFB9 NDUFC2 NDUFS1 NDUFS2 NDUFS3 NDUFS4 NDUFS6 NDUFS7 NDUFS8 NDUFV1 NDUFV2 NEPRO NEU1 NEUROD1 NFE2L2 NFS1 NFU1 NGLY1 NHLRC1 NME3 NMNAT1 NNT NOLA2 NOLA3 NPC1 NPC2 NPL NPM1 NR1H4 NR3C1 NR3C2 NSDHL NSUN2 NSUN3 NT5C3A NT5E NUBPL NUDT15 NUS1 OAS1 OAT OCRL ODC1 OGDH OGT OPA1 OPA3 OPLAH OSGEP OSTC OTC OXA1L OXCT1 PAH PAICS PAM16 PANK2 PAPSS2 PARN PARS2 PAX4 PC PCBD1 PCCA PCCB PCK1 PCK2 PCSK1 PCSK9 PCYT1A PCYT2 PDE12 PDHA1 PDHB PDHX PDK3 PDP1 PDPR PDSS1 PDSS2 PDX1 PDXK PDZK1IP1 PEPD PET100 PET117 PEX1 PEX10 PEX11B PEX12 PEX13 PEX14 PEX16 PEX19 PEX2 PEX26 PEX3 PEX5 PEX6 PEX7 PFKM PGAM2 PGAP1 PGAP2 PGAP3 PGK1 PGM1 PGM3 PGR PHGDH PHKA1 PHKA2 PHKB PHKG2 PHYH PHYKPL PI4K2A PI4KA PIGA PIGB PIGC PIGG PIGH PIGK PIGL PIGM PIGN PIGO PIGP PIGQ PIGS PIGT PIGU PIGV PIGW PIGY PIK3C2A PIK3CA PIK3CD PIK3R1 PIK3R2 PIK3R5 PIKFYVE PINK1 PIP5K1C PISD PITRM1 PKLR PLA2G4A PLA2G6 PLCB1 PLCB3 PLCB4 PLCD1 PLCE1 PLCG2 PLIN1 PLIN5 PLPBP PMM2 PMPCA PMPCB PMVK PNKD PNP PNPLA1 PNPLA2 PNPLA4 PNPLA6 PNPLA8 PNPO PNPT1 POFUT1 POGLUT1 POLG POLG2 POLR1A POLR1B POLR1C POLR1D POLR3A POLR3B POLR3H POLRMT POMGNT1 POMGNT2 POMK POMT1 POMT2 POP1 POR PPA2 PPCS PPM1K PPOX PPT1 PRDX1 PRKAG2 PRKCSH PRKN PRODH PRODH2 PRORP PRPS1 PRRT2 PSAP PSAT1 PSPH PSTPIP1 PTCD3 PTDSS1 PTEN PTRH2 PTS PUS1 PUS3 PYCR1 PYCR2 PYGL PYGM QARS1 QDPR QRSL1 RAB18 RAB23 RAB27A RAB3GAP1 RAB3GAP2 RAB7A RARS1 RARS2 RBCK1 RBSN RFT1 RFX6 RIC3 RMND1 RMRP RNASEH1 RNASEH2A RNASEH2B RNASEH2C RNASET2 RNF31 RPIA RPL10 RPL11 RPL13 RPL15 RPL18 RPL21 RPL26 RPL27 RPL35 RPL35A RPL5 RPS10 RPS15A RPS17 RPS19 RPS20 RPS23 RPS24 RPS26 RPS27 RPS28 RPS29 RPS7 RPSA RRM2B RTN4IP1 RUBCN RXYLT1 SACS SAMHD1 SAR1B SARDH SARS1 SARS2 SAT1 SBDS SBF1 SBF2 SC5D SCARB1 SCARB2 SCO1 SCO2 SCP2 SCYL1 SCYL2 SDHA SDHAF1 SDHAF2 SDHB SDHC SDHD SDR9C7 SEC23A SEC23B SECISBP2 SELENBP1 SELENOI SEPSECS SERAC1 SFXN4 SGMS2 SGPL1 SGSH SHMT2 SHPK SI SLC10A1 SLC10A2 SLC10A7 SLC11A2 SLC13A3 SLC13A5 SLC16A1 SLC17A5 SLC18A2 SLC19A1 SLC19A2 SLC19A3 SLC1A1 SLC1A2 SLC1A3 SLC1A4 SLC22A12 SLC22A5 SLC25A1 SLC25A10 SLC25A11 SLC25A12 SLC25A13 SLC25A15 SLC25A19 SLC25A20 SLC25A21 SLC25A22 SLC25A24 SLC25A26 SLC25A3 SLC25A32 SLC25A38 SLC25A4 SLC25A42 SLC25A46 SLC26A1 SLC26A2 SLC27A4 SLC27A5 SLC28A1 SLC29A1 SLC29A3 SLC2A1 SLC2A10 SLC2A2 SLC2A9 SLC30A10 SLC30A2 SLC30A9 SLC33A1 SLC35A1 SLC35A2 SLC35A3 SLC35C1 SLC35D1 SLC36A2 SLC37A4 SLC38A8 SLC39A13 SLC39A14 SLC39A4 SLC39A8 SLC3A1 SLC40A1 SLC45A1 SLC46A1 SLC52A1 SLC52A2 SLC52A3 SLC5A1 SLC5A2 SLC5A6 SLC5A7 SLC6A1 SLC6A17 SLC6A19 SLC6A2 SLC6A20 SLC6A3 SLC6A5 SLC6A6 SLC6A8 SLC6A9 SLC7A14 SLC7A2 SLC7A3 SLC7A5 SLC7A7 SLC7A9 SLC9A7 SLCO1B1 SLCO1B3 SLCO2A1 SMPD1 SMPD4 SMS SNAP25 SNAP29 SNORD118 SNX14 SORCS3 SORD SPATA5 SPG11 SPG20 SPG7 SPNS2 SPR SPTLC1 SPTLC2 SQOR SRD5A2 SRD5A3 SSBP1 SSR3 SSR4 ST3GAL3 ST3GAL5 STAP1 STAR STAT2 STEAP3 STS STT3A STT3B STX11 STX1B STXBP1 STXBP2 SUCLA2 SUCLG1 SUGCT SULT2B1 SUMF1 SUOX SURF1 SV2A SYN1 SYNJ1 SYT1 SYT14 SYT2 TACO1 TAF1A TALDO1 TANGO2 TARS1 TARS2 TAT TAZ TBC1D24 TBK1 TBXAS1 TCN1 TCN2 TCOF1 TDO2 TECPR2 TECR TF TFAM TFR2 TFRC TH THAP11 THG1L TIMM22 TIMM50 TIMM8A TIMMDC1 TK2

TKFC TKT TMEM126A TMEM126B TMEM165 TMEM173 TMEM199 TMEM70 TMLHE TMPRSS6 TOMM70 TOP3A TOR1A TP53RK TPI1 TPK1 TPMT TPP1 TPRKB TRAK1 TRAPPC11 TRAPPC12 TRAPPC2 TRAPPC2L TRAPPC4 TRAPPC6B TRAPPC9 TREH TREX1 TRIP11 TRIT1 TRMT1 TRMT10A TRMT10C TRMT5 TRMU TRNT1 TSEN15 TSEN2 TSEN34 TSEN54 TSFM TSR2 TTC19 TTPA TUFM TUSC3 TWNK TXN2 TXNRD2 TYMP TYR UBIAD1 UBTF UCP2 UGCG UGDH UGP2 UGT1A1 UMPS UNC13D UNG UPB1 UQCC2 UQCC3 UQCRB UQCRC2 UQCRFS1 UQCRQ UROC1 UROD UROS VAC14 VAMP1 VAMP2 VAPB VARS1 VARS2 VIPAS39 VKORC1 VLDLR VMA21 VPS11 VPS13A VPS13B VPS13C VPS13D VPS33A VPS33B VPS45 WARS1 WARS2 WDR4 WDR45 XDH XPNPEP3 XYLT1 XYLT2 YARS1 YARS2 YME1L1 YRDC ZFYVE26 ZNF143

CGH Array data

GLDC27-iPS4F-1 iPSCs			
Chromosome	*Start*	*End*	*CNV Value*
1	25598276	25647782	1
1	104109722	104309132	3
5	37006919	37024269	3
6	29094696	29161435	1
10	123343892	123344530	1
10	123436246	123461066	1
12	33302438	33305855	0
16	572044	783004	3
17	44265839	44268953	3
17	52156400	52169120	0

GLDC deficient fibroblasts (original patient's fibroblasts)			
Chromosome	*Start*	*End*	*CNV Value*
1	25598276	25647782	1
-	-	-	-
5	37006919	37017880	3
6	29094696	29161435	1
10	123343892	123344440	1
10	123436246	123461066	1
12	33302438	33305855	0
16	572044	865040	3
17	44267617	44268953	4
17	52156400	52169120	0

All events in the GLDC27-iPS4F-1 iPSCs line were detected in the original patient's fibroblasts, except for an event on chromosome 1 that spans from position 104109722 to position 104309132. However, this event appeared to be in a region with minimal SNPs and, therefore, a low coverage area due to hybridization specific to the probes used in the analysis. The region encompassed by this event does not encompass relevant gene sequences.
