# Supplementary material for: Metabolic Rewiring and Altered Glial Differentiation in an iPSC-Derived Astrocyte Model Derived from a Nonketotic Hyperglycinemia Patient
Source: Int J Mol Sci. 2024 Feb 28;25(5):2814. doi: 10.3390/ijms25052814 (PMC10931996; doi:10.3390/ijms25052814)
Supplement: Supplementary file 1 [file ijms-25-02814-s001.zip › Supplementary Table S1_L.pdf]

Table S1: **Primers sequence**

Gene	Genbank Ref.	Protein Name	Sequence 5'-3'
GLDC	NM_000170.3	Glycine decarboxylase	TGGCATGGGCTATTATAACT<br>GTATGGAGTATACTGGGTGATCC
GCSH	NM_004483.5	Glycine cleavage system protein H	GCATTGGAACAGTGGGAAT<br>CCAACTTCAGGGAGACTACAATA
AMT	NM_000481.4	Aminomethyltransferase	AGGAGGTGCTCCGCAGGA<br>GCAGCATATGAGACACGTCAAAG
PHGDH	NM_006623.4	Phosphoglycerate dehydrogenase	CAGCACCAAGGAGGCTCA<br>CAGCAATTTCCTCCCCACA
PSAT1	NM_058179.4	Phosphoserine aminotransferase 1	AGTGGAGCCCCAAAATAGAA<br>CCTATGCCCTTTCAAGGAC
PSPH	NM_004577.4	Phosphoserine phosphatase	AGGAAGCTTTTCTACTCAGCA<br>GAGTCTCTGCACCTGCTC
SHMT1	NM_004169.5	Serine hydroxymethyltransferase 1	GACTGAGTTTATTGATGAACTGG<br>CCTGAGTAGGGCTGGAC
SHMT2	NM_005412.6	Serine hydroxymethyltransferase 2	TCAGCCACGTCCATCTTCT<br>CAGTGCCAGCTGGTTGTAGT
SRR	NM_021947.3	Serine racemase	GCAATACAAGCCTACGGAGC<br>GGCAATTGTCCCTTGTCCAG

Folate Cycle

Gene	Genbank Ref.	Protein Name	Sequence 5'-3'
MTHFD1	NM_005956.4	Methylenetetrahydrofolate dehydrogenase 1	TTGGACAGGCTCCAACGGAGAA<br>AGAAGTGGTGAGAGCCAGGACA
MTHFD2	NM_006636.4	Methylenetetrahydrofolate dehydrogenase 2	CTCCTTGTTCAGTTGCCTCTTCC<br>CTGATCCAAACACATTCGTCCTAC
MTHFR	NM_005957.5	Methylenetetrahydrofolate reductase	AGATGGCTACCACAGAGGTGCT<br>TGGGACGTACATCTTCCTCTCG
DHFR	NM_000791.4	Dihydrofolate reductase	CATGGTCTGGATAGTTGGTGGC<br>GTGTCACTTTCAAAGTCTTGCATG
TYMS	NM_001071.4	Thymidylate synthetase	GGTGTTTTGGAGGAGTTGCTGTG<br>GGAGAATCCCAGGCTGTCCAAA

Creatine Cycle

Gene	Genbank Ref.	Protein Name	Sequence 5'-3'
AGAT	NM_001482.3	Glycine amidinotransferase	CCCACCACCTGTTCCCGGC<br>GCATATATCACAATGTTGAGATG

mtDNA Depletion

Gene	Genbank Ref.	Protein Name	Sequence 5'-3'
ND1	NM_173708.1	NADH dehydrogenase 1	GGCTATATACAACTACGCAAAGGC<br>GGTAGATGTGGCGGGTTTTAGG
12S (MT-RNR1)	NC_012920.1	Mitochondrially encoded 12S rRNA	TGGCCACAGCACTTAAACAC<br>TGGTTAGGCTGGTGTTAGGG
18S	NR_003286	Mitochondrially encoded 18S rRNA	ATCCATTGGAGGGCAAGTC<br>GCTCCCAAGATCCAACTACG

Cell Cycle

Gene	Genbank Ref.	Protein Name	Sequence 5'-3'
CCNA2	NM_001237.5	Cyclin A2	CTCTACACAGTCACGGGACAAAG<br>CTGTGGTGCTTTGAGGTAGGTC
CCND1	NM_053056.3	Cyclin D1	TCTACACCGACAACTCCATCCG<br>TCTGGCATTTTGGAGAGGAAGTG
CDK1	NM_001786.5	Cyclin-dependent kinase 1	GGAAACCAGGAAGCCTAGCATC<br>GGATGATTCAGTGCCATTTTGCC
CDK2	NM_001798.5	Cyclin-dependent kinase 2	ATGGATGCCTCTGCTCTCACTG<br>CCCGATGAGAATGGCAGAAAGC

NPCs Characterization

Gene	Genbank Ref.	Protein Name	Sequence 5'-3'
LIN28B	NM_024674.6	Lin-28 homolog B	GGCATCTGTAAGTGGTTCAACG<br>CCTCCTTCAAGCTCCGGA
OCT3/4	NM_001173531.3	POU class 5 homeobox 1	AGTTTGTGCCAGGGTTTTTG<br>ACTTCACCTTCCCTCCAACC
PAX6	NM_001368894.2	Paired box 6	AATAACCTGCCTATGCAACCC<br>AACTTGAACTGGAACTGACACAC
NES	NM_006617.2	Nestin	TGGCAAAGGAGCCTACTCCAAGAA<br>ATCGGGATTCAGCTGACTTAGCCT
SOX1	NM_005986.3	SRY-box transcription factor 1	ATGCACCGCTACGACATGG<br>CTCATGTAGCCCTGCGAGTTG
MAP2	NM_001375505.1	Microtubule-associated protein 2	AAAGCTGATGAGGGCAAGAA<br>GGCCCCTGAATAAATTCCAT
FOXG1	NM_005249.5	Forkhead box G1	TACTACCGCGAGAACAAGCA<br>TCACGAAGCACTTGTTGAGG

iAs Characterization

Gene	Genbank Ref.	Protein Name	Sequence 5'-3'
S100β	NM_006272.3	S100 calcium binding protein B	TGTAGACCCTAACCCGGAGG<br>TGCATGGATGAGGAACGCAT

GFAP	NM_002055.5	Glial fibrillary acidic protein	GTCCCCCACCTAGTTTGCAG<br>TAGTCGTTGGCTTCGTGCTT
VIM	NM_003380.5	Vimentin	TGGACCAGCTAACCAACGAC<br>GCCAGAGACGCATTGTCAAC
ALDH1L1	NM_001270364.2	Aldehyde dehydrogenase 1 family member L1	GGATGCCTTTGAGAATGGACGG<br>TCCTGGTGCTGCTCCATGAGAT
AQP4	NM_001317384.3	Aquaporin 4	GCCATCATTGGAGCAGGAATCC<br>ACTCAACCAGGAGACCATGACC
APOE	NM_000041.4	Apolipoprotein E	GGGTCGCTTTTGGGATTACCTG<br>CAACTCCTTCATGGTCTCGTCC
GLAST	NM_001166695.3	Solute carrier family 1 member 3	TAAAGTGCCCATCCAGGC<br>AAGACAACTAGACCCAGGGC
GLT-1	NM_006516.4	Solute carrier family 2 member 1	CATGGGCTTCTCGAAACTG<br>AAGGGCTGTGGGTGACAC
MAP2	NM_001375505.1	Microtubule associated protein 2	AAAGCTGATGAGGGCAAGAA<br>GGCCCCTGAATAAATTCCAT
NeuN	NM_001350451.2	RNA binding fox-1 homolog 3	TACGCAGCCTACAGATACGCTC<br>TGGTTCCAATGCTGTAGGTCGC

iAs Metabolism

Gene	Genbank Ref.	Protein Name	Sequence 5'-3'
GLYT1	NM_001024845.3	Solute carrier family 6 member 9	GTCACAGCCATTGTGGATGAGG<br>GCCGCATAGTTGTCCATCAGCA

GLYT2	NM_004211.5	Solute carrier family 6 member 5	CTGATGCTCCTCACTCTTGGAC<br>TGCGTAGGTACTTGGGAAACTCG

Endogenous Control

Gene	Genbank Ref.	Protein Name	Sequence 5'-3'
ACTB	NM_001101.5	Actin beta	GGCATGGGTCAGAAGGATTC<br>CACAGCGAGCTCATTGTAGAAG
TUBB1	NM_030773.4	Tubulin beta	CTTTGTGGAATGGATCCCCA<br>GACTGCCATCTTGAGGCCA
