# Supplementary material for: Metabolic Rewiring and Altered Glial Differentiation in an iPSC-Derived Astrocyte Model Derived from a Nonketotic Hyperglycinemia Patient
Source: Int J Mol Sci. 2024 Feb 28;25(5):2814. doi: 10.3390/ijms25052814 (PMC10931996; doi:10.3390/ijms25052814)
Supplement: Supplementary file 1 [file ijms-25-02814-s001.zip › Supplementary Table S2_L.pdf]

Table S2: Antibodies

Antibody	Protein	Host	Use	Reference	Company
**Anti-AGAT**	Glycine amidinotransferase	Rabbit, pAb	WB (1:1000)	12801-1-AP	Proteintech
**Anti-CV-ATP5α**	ATP synthase alfa subunit	Mouse, mAb	WB (1:1000) IF (1:500)	ab14748	Abcam
**Anti-GAPDH**	Glyceraldehyde-3-phosphate dehydrogenase	Mouse, mAb	WB (1:5000)	ab8245	Abcam
**Anti-GCSH**	Glycine cleavage system protein H	Rabbit, pAb	WB (1:1000); IF (1:500)	16726-1-AP	Proteintech
**Anti-GCSP**	Glycine decarboxylase	Mouse, pAb	WB (1:2000) IF (1:200)	NBP1-32907	Novus Biological
**Anti-GCST**	Aminomethyltransferase	Mouse, pAb	WB (1:500)	NBP1-89783	Novus Biological
**Anti-GFAP**	Glial fibrillary acidic protein	Mouse, mAb	IF (1:50)	3670S	Cell Signalling
**Anti-GLAST1**	Solute carrier family 1 member 3	Rat, pAb	IF (1:40)	-	-
**Anti-GLT-1**	Solute carrier family 2 member 1	Rat, pAb	IF (1:40)	-	-
**Anti-MAP2**	Microtubule-associated protein 2	Mouse, mAb	IF (1:100)	M1406	Sigma Aldrich
**Anti-Nestin**	Nestin	Mouse, mAb	IF (1:100)	ab18102	Abcam
**Anti-OCT3/4**	POU class 5 homeobox 1	Mouse, mAb	IF 1:60	sc-5279	Santa Cruz Biotechnolog y
**Anti-OxPhos**	CI-NDUFB8, CII-SDHB, CIII-UQCRC1, CIV-MTCOI, CV-ATP5A	Mouse, mAb	WB (1:500)	ab110413	Abcam

**Anti-PAX6**	Paired box 6 protein	Rabbit, pAb	IF (1:100)	GTX113241	GeneTex
**Anti-PHGDH**	Phosphoglycerate dehydrogenase	Rabbit, pAb	WB (1:1000)	14719-1-AP	Proteintech
**Anti-PSAT1**	Phosphoserine aminotransferase 1	Rabbit, pAb	WB (1:1000)	10501-1-AP	Proteintech
**Anti-PSPH**	Phosphoserine phosphatase	Rabbit, pAb	WB (1:1000)	14513-1-AP	Proteintech
**Anti-S100β**	S100 calcium-binding protein β	Rabbit, mAb	IF (1:50)	ab52642	Abcam
**Anti-SHMT1**	Serine hydroxymethyltransferase 1	Rabbit, pAb	WB (1:1000)	14149-1-AP	Proteintech
**Anti-SHMT2**	Serine hydroxymethyltransferase 2	Rabbit, pAb	WB (1:1000)	11099-1-AP	Proteintech
**Anti-SRR**	Serine racemase	Rabbit, pAb	WB (1:1000)	17955-1-AP	Proteintech

Secondary Antibodies

Antibody	Protein	Host	Use	Reference	Company
**Alexa 488 Anti-rabbit-IgG**	Rabbit IgG	Donkey	IF 1:500	A-21206	ThermoFisher
**Alexa 555 Anti-mouse-IgG**	Mouse IgG	Donkey	IF 1:500	A-31570	ThermoFisher
**Anti-rabbit-IgG-HRP**	Rabbit IgG	Goat	WB, BN-WB (1:5000)	7074S	Cell Signalling
**Anti-mouse-IgG-HRP**	Mouse IgG	Horse	WB, BN-WB (1:2000)	7076S	Cell Signalling

WB: Western blot; **IF:** Immunofluorescence; **BN-WB:** Blue Native Western blot; **pAb:** Polyclonal antibody; **mAb:** Monoclonal antibody
